# Combatting Substandard and Falsified Medicines: A View from Rwanda

**DOI:** 10.1371/journal.pmed.1001476

**Published:** 2013-07-02

**Authors:** Agnes Binagwaho, Roger Bate, Michel Gasana, Corine Karema, Yves Mucyo, John Patrick Mwesigye, Floribert Biziyaremye, Cameron T. Nutt, Claire M. Wagner, Paul Jensen, Amir Attaran

**Affiliations:** 1 Ministry of Health of Rwanda, Kigali, Rwanda; 2 Harvard Medical School, Boston, Massachusetts, United States of America; 3 Geisel School of Medicine at Dartmouth, Hanover, New Hampshire, United States of America; 4 American Enterprise Institute, Washington, District of Columbia, United States of America; 5 Rwanda Biomedical Center, Kigali, Rwanda; 6 Dartmouth Center for Health Care Delivery Science, Hanover, New Hampshire, United States of America; 7 Global Health Delivery Partnership, Boston, Massachusetts, United States of America; 8 Pivit, Washington, District of Columbia, United States of America; 9 University of Ottawa, Ottawa, Ontario, Canada

## Abstract

Agnes Binagwaho and colleagues describe Rwanda's experience of pharmacovigilance for malaria and tuberculosis, and call for a global treaty and leadership by the World Health Organization to address the global manufacture and trade in substandard and falsified medicines.

*Please see later in the article for the Editors' Summary*

**Summary Points**

- Substandard and falsified medicines are major global health challenges that cause unnecessary morbidity and mortality around the world and threaten to undermine recent progress against infectious diseases by facilitating the emergence of drug resistance.
- In a recent study, Rwanda had the lowest prevalence of poor quality tuberculosis drugs among African countries in the sample. This positive finding may be associated with Rwanda's efforts to ban the sale of monotherapies, ensure that private sellers of important medicines are qualified, and prioritize the prevention of falsified medicines entering the country.
- Drawing on our experiences in Rwanda scaling up pharmacovigilance for malaria and tuberculosis, we call for a global treaty and leadership by the World Health Organization to address manufacturing and trade in substandard and falsified medicines.

## A Global Threat

In the last year, several institutions—the World Health Organization (WHO), the United Nations Office on Drugs and Crime, and the United States Institute of Medicine—have turned their attention, with varying degrees of effectiveness, to the problem of dangerously poor quality medicines [1]. While substandard medicines are found everywhere in the world, it is the poorest countries with the weakest capacity for drug regulation and quality control that suffer the most.

Everyone agrees that poor quality medicines are undesirable, but not everyone agrees on how to define them. It is clear that iatrogenic harm arises from at least two distinct, concurrent problems: (i) medicines that are accidentally or negligently “substandard” as a result of various failures in manufacturing, handling, regulation, or some combination of these, and (ii) medicines that are deliberately “falsified,”—neither being of the correct standard nor being properly registered through a country's regulatory authority—and that call out for criminal law measures to suppress. In all cases, the result is a potentially dangerous medicine, whether occasioned by criminal activity, accident, or negligence [2].

A sound drug regulatory system is essential to overcome these problems. Yet while WHO estimates that nearly a third of its member states have very limited or no capacity for medicine regulation [3], it has yet to propose a concrete action plan to help ensure the quality of national drug supplies [2].

## Undermining Tuberculosis Control

While the malaria community has worked since the 1990s to address the problem of poor quality drugs, the tuberculosis control community has come to it late [4]. The available evidence suggests a widespread problem with substandard and falsified anti-tuberculosis drugs available in low- and middle-income countries, but little awareness that such a threat exists.

The global scope of the danger is strongly hinted at by a study conducted by some of the authors of this Essay [4]. Among 713 samples of isoniazid and rifampicin purchased at community pharmacies in 19 cities of 17 countries, 65 (9.1%) of the samples had insufficient active pharmaceutical ingredient (API) and failed basic quality control tests. Of those samples 18 (of 65, or 27.7%) were definitely falsified, since they had zero API and obviously fake packaging. The remaining products with insufficient API (47 of 65, or 72.3%) were either substandard or falsified; there is not evidence enough to determine which, because these products could have been faulty by accident (the hallmark of substandard drugs) or faulty by criminal intent (the hallmark of falsified drugs), although both are dangerous. Certainly, the most dangerous failures are the 36 (55.4%) products that contained more than trace amounts of isoniazid and rifampicin, and that had an intermediate dose (between 10% and 80%) likely insufficient to cure disease, but definitely capable of selecting the drug-resistant bacilli that cause life-threatening multidrug-resistant and extensively drug-resistant tuberculosis. The situation is even worse in the study's 11 African countries, where 27 of 163 total samples (16.6%) failed testing. Fifteen of these 27 failures (44.4%) were probably substandard drugs, while at least 12 (55.6%) were falsified drugs. These findings were in line with previous studies [5],[6].

## Rwanda's Efforts to Ensure Drug Quality

Rwanda was the only African nation whose samples did not include any falsified products. Rwanda has also performed better than other African nations in other large studies, having few substandard and no obviously falsified products [4],[7],[8]. There are two probable reasons for this success: first, the government of Rwanda has taken concrete legal and technical steps to ensure the quality of its drug supply chain; second, Rwanda was able to do that because it brought tuberculosis control into the publicly funded health system to a greater extent than many countries, which often rely on unregulated treatment in larger private sectors.

Aware of the dangers of poor quality drugs for tuberculosis and malaria, in the late 1990s Rwanda mandated that all drug contracts awarded by the Ministry of Health must be to manufacturers with current WHO-approved certificates of Good Manufacturing Practices. Recent research on artemisinin combination therapies (ACTs) for malaria has shown that products of WHO-approved manufacturers are up to five times less likely to fail basic quality tests [9].

Rwanda's “Guidelines for Pharmacovigilance and Medicine Information System in Rwanda” [10] were adopted in early 2011, and led to the formation of pharmacovigilance sub-committees at all 469 health centers that are overseen by the country's 42 district hospitals. Standardized notification forms for poor quality health products are available to patients, community health workers, nurses, physicians, and pharmacists at health facilities, where they are collected for periodic review by the National Pharmacovigilance and Medicine Information Center in the Ministry of Health. To date, more than 2,400 health workers have been trained in the implementation of the guidelines.

With the support of national and international partners, the Ministry of Health conducts quality control by testing each imported drug shipment as well as systematically sampling ACTs and anti-tuberculosis drugs from each level of the health sector on a quarterly and annual basis, respectively. These medicines are distributed via a dedicated supply chain including climate-controlled storage facilities and vehicles.

Rwanda has also largely succeeded in prohibiting sales of malaria monotherapies and all anti-tuberculosis drugs through private pharmacies, where the provision of monotherapies for both diseases poses a major global regulatory challenge (both infections respond best to combination therapy). Private health facilities, however, can continue to provide tuberculosis treatment if they become accredited as Centers for Diagnosis and Treatment of Tuberculosis, which are integrated into the public sector supply chain and provide quality-assured drugs for free to tuberculosis patients. Private pharmacies can sell ACTs to patients who have a prescription from a health worker following a positive diagnostic test.

Rwanda's Bureau of Standards verifies certification documents and, together with the Customs Services Department and the Ministry of Health, inspects each shipment of drugs upon importation into the country. Where falsified medicines are found, these authorities partner with the Rwandan police force and Interpol to denounce and to try to arrest the organized criminals responsible. Thus, Rwanda's drug quality control strategy depends not only on its health system, but on its law enforcement and justice systems as well.

## Positive Signs and Next Steps

As policymakers in, and researchers of, Rwanda's health sector, we argue that the improvement of the country's supply chain and drug surveillance systems, combined with equity-oriented strategies for increasing geographic and financial access to high quality drugs through the public sector, has played an important role in the country's steep declines in mortality due to tuberculosis and malaria. Between 2000 and 2011, Rwanda's tuberculosis mortality rate declined 77.1%, and reported annual malaria deaths dropped 85.3% from 2005 to 2011 [11]. Treatment success rates for new smear-positive cases of drug-susceptible tuberculosis increased from 63% in 2000 to 88% in 2010 [12]. Further, 88% of patients treated for multidrug-resistant tuberculosis through 2011 had a successful outcome [13], and the country has not yet seen a case of extensively drug-resistant tuberculosis.

Rwanda understands that the problems of falsified and substandard drugs cannot be effectively addressed by one institution or one country working alone. It is therefore contributing to the drafting of a regional law against falsified medicines with other member states of the East African Community, and plans to advocate for clear language distinguishing between generic and falsified medicines. Rwanda is also a party to the recently launched East African Community Medicines Regulatory Harmonization Initiative.

## Time for Action

It is time for the tuberculosis control community to recognize the causes and consequences of falsified and substandard drugs and to mobilize a global response. Evidence-driven actions at the national level are essential and should be supported by multilateral donors, but will not be sufficient to solve this global problem.

We propose that a global treaty is now needed to guarantee sustainable progress towards higher quality medicines by bringing regulatory, technical, legal, and financial mechanisms to bear together [14]. Such a treaty will be possible only with visionary and transparent leadership from WHO on behalf of its member states [2]. As the dangers of drug-resistant tuberculosis fed by poor quality medicines illustrate all too well, the world loses ground with each passing day that WHO delays.

## Abbreviations

ACT: artemisinin combination therapy
API: active pharmaceutical ingredient
WHO: World Health Organization
